# Distended abdomen due to a pseudocyst around a ventriculoperitoneal shunt

**DOI:** 10.1007/s10354-021-00870-6

**Published:** 2021-08-02

**Authors:** Sascha Meyer, Sogand Nemat, Stefan Linsler, Johannes Bay, Michael Zemlin, Martin Poryo

**Affiliations:** 1 Building 9, Department of Pediatrics and Neonatology, University Medical Center of Saarland, 66421 Homburg, Germany; 2 Department of Radiology, University Medical Center of Saarland, Homburg, Germany; 3 Department of Neurosurgery, University Medical Center of Saarland, Homburg, Germany; 4 Department of Pediatric Cardiology, University Medical Center of Saarland, Homburg, Germany

**Keywords:** Prematurity, Ventriculo-peritoneal shunt, Abdomen, Cyst, Ultrasonography

## Abstract

Described herein is a case of distended abdomen in a 4-year-old boy with a ventriculoperitoneal (VP) shunt due to bilateral intraventricular hemorrhage following premature birth. Physical examination and laboratory tests revealed tenderness in the lower quadrants, with mild leukocytosis and normal C‑reactive protein levels. X‑ray demonstrated an intact VP shunt catheter but cranial displacement of the large intestine. Ultrasonography confirmed a large pseudocyst around the VP shunt, with extension from the symphysis to the sternum. The distal part of the VP shunt was surgically revised and 2.5 l of cerebrospinal fluid were evacuated. The boy made a full clinical recovery. Conventional X‑rays, routinely used to confirm or exclude VP shunt continuity, may provide important clues regarding to the etiology of VP shunt dysfunction.

## Case report

This case report describes a 4-year-old boy with a history of bilateral intraventricular hemorrhage requiring the insertion of a permanent ventriculoperitoneal (VP) shunt after being born as premature infant after 28 weeks of gestation. Upon admission to our hospital, the parents reported recurrent vomiting, but no fever or diarrhea.

On physical examination he looked unwell, and on palpation the abdomen was distended and demonstrated tenderness in both lower quadrants. The VP shunt could be easily palpated, with no signs of discontinuation. Laboratory analysis showed mild leukocytosis but C‑reactive protein within the reference range.

**Fig. 1:**
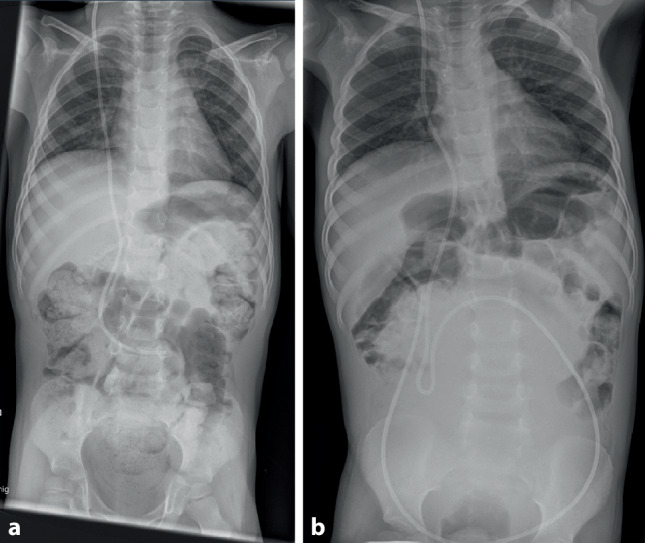
**a** X-ray demonstrating cranial displacement of the large intestine, **b** X-ray demonstrating regular intra-abdominal gas pattern

To rule out VP shunt disconnection, an X‑ray study was performed demonstrating an intact VP shunt catheter but cranial displacement of the large intestine (Fig. 1a), most notably when compared to a previous examination (Fig. 1b; with a different VP shunt).

**Fig. 2:**
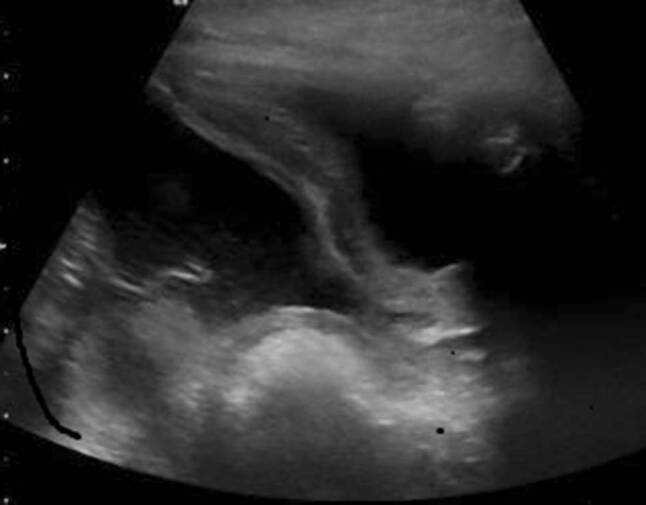
Ultra-sonography showing large intra-abdominal pseudocyst

On ultrasonography, a large pseudocyst around the VP shunt was confirmed, with extension from the symphysis to the sternum (Fig. 2). Subsequently, the distal part of the VP shunt was surgically revised and 2.5 l of cerebrospinal fluid were evacuated. After surgical revision, the boy made a full clinical recovery.

We conclude that conventional X‑rays—although routinely used to confirm or exclude VP shunt continuity—may provide important clues with regard to the etiology of VP shunt dysfunction [1, 2].
